# Nanoscale Surface Topography Modulates hIAPP Aggregation Pathways at Solid–Liquid Interfaces

**DOI:** 10.3390/ijms22105142

**Published:** 2021-05-13

**Authors:** Marcel Hanke, Yu Yang, Yuxin Ji, Guido Grundmeier, Adrian Keller

**Affiliations:** Technical and Macromolecular Chemistry, Paderborn University, Warburger Str. 100, 33098 Paderborn, Germany; marcelha@mail.uni-paderborn.de (M.H.); yuyang@mail.uni-paderborn.de (Y.Y.); jiyuxin777@gmail.com (Y.J.); g.grundmeier@tc.uni-paderborn.de (G.G.)

**Keywords:** surface topography, amyloid, adsorption, atomic force microscopy, pattern formation, self-assembly

## Abstract

The effects that solid–liquid interfaces exert on the aggregation of proteins and peptides are of high relevance for various fields of basic and applied research, ranging from molecular biology and biomedicine to nanotechnology. While the influence of surface chemistry has received a lot of attention in this context, the role of surface topography has mostly been neglected so far. In this work, therefore, we investigate the aggregation of the type 2 diabetes-associated peptide hormone hIAPP in contact with flat and nanopatterned silicon oxide surfaces. The nanopatterned surfaces are produced by ion beam irradiation, resulting in well-defined anisotropic ripple patterns with heights and periodicities of about 1.5 and 30 nm, respectively. Using time-lapse atomic force microscopy, the morphology of the hIAPP aggregates is characterized quantitatively. Aggregation results in both amorphous aggregates and amyloid fibrils, with the presence of the nanopatterns leading to retarded fibrillization and stronger amorphous aggregation. This is attributed to structural differences in the amorphous aggregates formed at the nanopatterned surface, which result in a lower propensity for nucleating amyloid fibrillization. Our results demonstrate that nanoscale surface topography may modulate peptide and protein aggregation pathways in complex and intricate ways.

## 1. Introduction

The adsorption of proteins and peptides at solid–liquid interfaces is frequently encountered in numerous research fields, ranging from biology and medicine to analytical chemistry and materials science. Relevant examples include microbial adhesion and biofilm formation [1], tissue integration of implants [2], nanoparticle-based drug delivery [3], biomarker detection in blood samples [4], and the synthesis of biocompatible functional thin films [5]. Since protein adsorption is governed by a complex interplay of electrostatic, van der Waals, and hydrophobic interactions, as well as hydrogen and sometimes even covalent [6] bond formation, the physicochemical properties of the surface are major modulating factors [7]. After decades of research, we have now obtained a rather consistent, albeit somewhat crude, picture of the effects of surface chemistry, wettability, and charge on protein adsorption kinetics and adsorption-induced protein denaturation [7]. In contrast, the role of surface topography and, in particular, nanotopography in protein and peptide adsorption is still rather elusive. Even though the effects of different nanoscale surface topographies on the adsorption of various proteins have been investigated in numerous studies [8,9,10,11,12,13,14,15,16,17,18,19,20], the underlying mechanisms are still not yet fully understood. This particularly concerns the question of how surface topography may affect also protein–protein interactions and protein aggregation during adsorption [14,21,22,23,24].

Protein and peptide aggregation is a ubiquitous phenomenon in biological systems. In many cases, the survival of the organism critically relies on such functional protein aggregates, which include, for instance, the filaments of the cytoskeleton [25], the fibers of the extracellular matrix [26], and the fibrils providing structural support in microbial biofilms [27]. However, protein aggregates may also be key players in the development of degenerative diseases. This particularly concerns so-called amyloid aggregates, which are composed of misfolded proteins and peptides and stabilized by intermolecular β-sheets [28]. Such pathogenic amyloid aggregates can have various morphologies, ranging from small particle-like oligomers to thin protofibrils to highly polymorphic mature fibrils that are formed by the lateral interaction of different numbers of protofibrils. When interacting with cells, these amyloid aggregates can damage the plasma membrane and thereby induce cell death [29]. Most importantly, a multitude of different proteins and peptides were identified that may aggregate into cytotoxic amyloids in vitro and sometimes also in vivo [30]. However, this often requires the destabilization of the native protein structure, for instance, by non-physiological pH, ionic strength, temperature, or monomer concentration. While it was already shown thirty years ago that the presence of interfaces can also stimulate protein and peptide aggregation [31], surface- and interface-related effects have only recently become a focus of attention [32,33,34]. This recent development was mostly motivated by the potential of nanoparticle-based strategies to mitigate amyloid-associated diseases [35,36,37,38,39,40,41,42,43], as well as by promising applications of amyloid fibrils in materials’ synthesis [44,45,46,47,48]. Nevertheless, the manifold effects of the physicochemical surface properties on amyloid aggregation and the involved molecular mechanisms are far from understood [33,34]. 

While several studies have investigated the influence of surface chemistry and wettability on amyloid aggregation [49,50,51,52,53,54,55,56,57,58,59,60,61,62], there are only a few publications so far that have addressed the effect of surface topography [23,24,62]. Shezad et al. investigated the aggregation of the Alzheimer’s-associated peptide Aβ(1–42) at hydrophobic polystyrene (PS) surfaces with random rough surface topographies [23]. They found that an increase in surface roughness resulted in the retardation or even complete inhibition of Aβ(1–42) fibrillization. This was explained by the rougher surfaces hindering the lateral diffusion of adsorbed peptide monomers along the surface, which prevented them from reaching and attaching to the ends of growing amyloid fibrils. This was recently verified by Co and Li in Monte Carlo simulations [24]. In this work, we investigate the effect of nanoscale surface topography on the aggregation of human islet amyloid polypeptide (hIAPP) at hydrophilic silicon oxide surfaces. The silicon oxide surface is negatively charged under physiological conditions [63] and can, thus, be considered a crude model of biological surfaces such as cell membranes [64] or blood vessel walls [65]. hIAPP is a 37-residue polypeptide hormone produced in the pancreas, whose aggregation is a key intermediate step in the development of type 2 diabetes mellitus [66] and is thus frequently studied in vitro, both in bulk solution [67,68,69,70,71,72,73,74,75,76] and in contact with solid surfaces [36,49,55,77,78,79]. Since random rough surfaces are difficult to describe and may exhibit very different morphologies despite identical roughness parameters [16], in the present work, we instead employed ion beam nanopatterning to produce silicon oxide surfaces with a well-defined and anisotropic nanoscale topography [80]. Using time-lapse atomic force microscopy (AFM) to characterize the evolution of hIAPP aggregate morphology quantitatively, we show that the presence of such nanopatterns modulates the hIAPP aggregation pathway. In particular, it appears that the nanopatterned surface retards hIAPP fibrillization compared to the flat surface while simultaneously promoting the formation of amorphous hIAPP aggregates.

## 2. Results

### 2.1. Characterization of the Flat and Nanopatterned Surfaces

**Figure 1:**
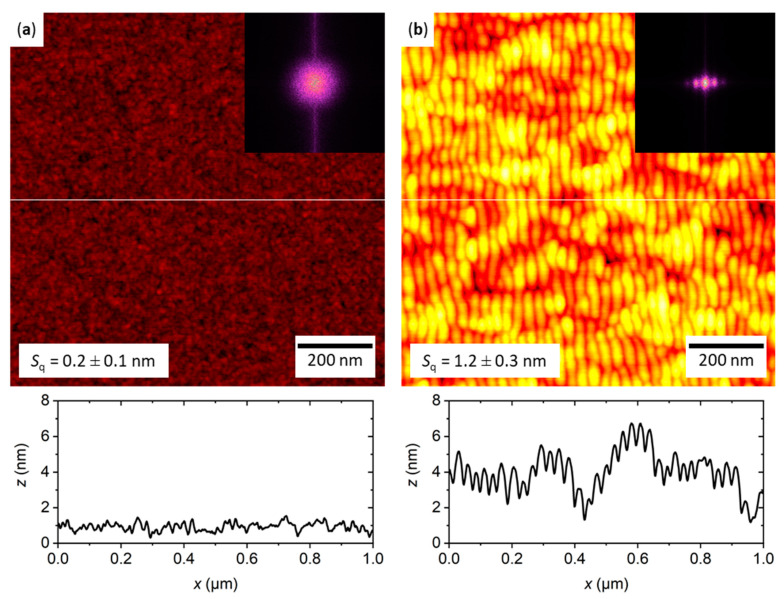
Representative AFM images of the oxidized silicon surfaces employed in this work. (**a**) The surface of a comparatively smooth untreated Si(100) wafer with a root mean square (RMS) surface roughness of *S*_q_ = 0.2 ± 0.1 nm. (**b**) After ion irradiation, the silicon surface displays a self-organized ripple pattern and the RMS roughness is increased to *S*_q_ = 1.2 ± 0.3 nm. Both images have the same height scale ranging from 0 to 8 nm. The insets show the corresponding FFTs of the images. Below each AFM image, a height profile recorded along the white horizontal line is shown.

Figure 1 shows topographic AFM images with corresponding height profiles of the two types of model surface employed in this work. An untreated silicon wafer with native surface oxide was chosen as the flat surface (Figure 1a). Its surface topography is comparatively smooth, with an average root mean square (RMS) surface roughness of *S*_q_ = 0.2 ± 0.1 nm. Nanopatterning of such flat silicon surfaces was performed using 500 eV Ar^+^ irradiation under oblique incidence, which induces the self-organized formation of regular nanoscale ripple patterns. The ripple pattern shown in Figure 1b is characterized by a quasi-sinusoidal topographic modulation with a periodicity and a peak-to-peak amplitude of 29.3 ± 4.3 and 1.5 ± 0.6 nm, respectively, as determined from the one-dimensional height difference correlation function [81]. This modulation results in well-defined correlation peaks in the two-dimensional fast Fourier transform (FFT) in the direction of the wave vector of the ripples (see the inset of Figure 1b). In contrast, the FFT of the flat surface is completely isotropic (see the inset of Figure 1a). Closer inspection of the AFM image and the corresponding height profile in Figure 1b reveals the presence of an underlying long-range roughness that causes additional height variations over length scales of hundreds of nanometers. In combination with the ripple pattern, this topographic height variation results in an increased surface roughness of *S*_q_ = 1.2 ± 0.3 nm. Most importantly, while Ar^+^ irradiation under the applied conditions leads to an increased thickness of the native oxide layer, it does not cause any significant changes in the chemical composition or the oxidation state of the oxide layer, as verified by X-ray photoelectron spectroscopy [15]. Therefore, any observed differences in hIAPP aggregation can be attributed to the differences in surface topography.

### 2.2. hIAPP Aggregation in Bulk Solution

**Figure 2:**
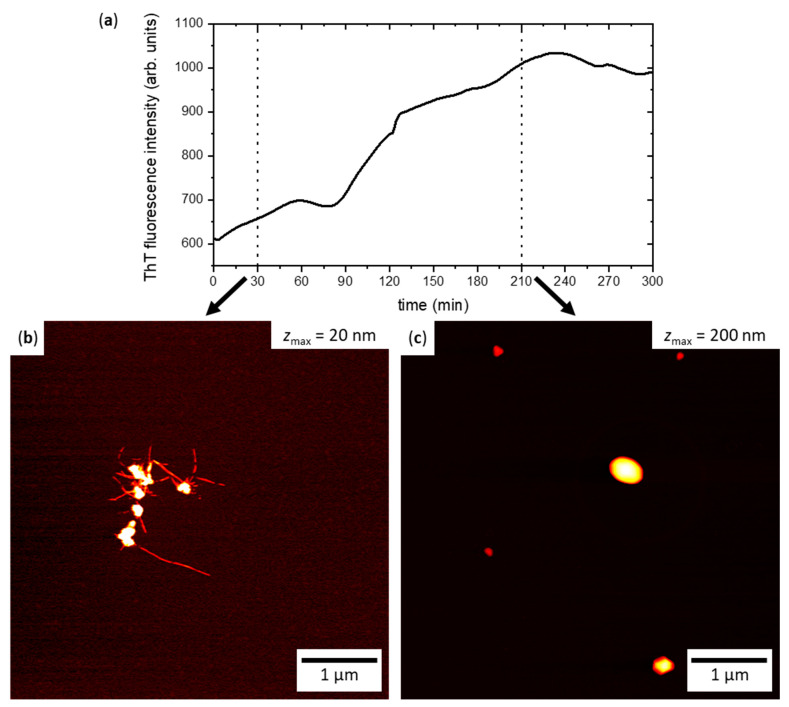
hIAPP aggregation in bulk solution. (**a**) ThT fluorescence intensity as a function of time. The plotted curve represents the average of three independent measurements. (**b**) AFM image of hIAPP aggregates obtained after 30 min incubation in bulk solution without ThT. (**c**) AFM image of hIAPP aggregates obtained after 210 min incubation in bulk solution without ThT. The height scales of the images in (**b**) and (**c**) range from 0 nm to *z*_max_, with *z*_max_ given in the images. See Figure S2 for additional AFM images.

The aggregation of hIAPP in bulk solution was investigated to provide a baseline for comparison with the results obtained in contact with the flat and nanopatterned surfaces. To this end, 2 µM hIAPP in phosphate-buffered saline (PBS, pH 7.4) was incubated at room temperature for 300 min. Amyloid aggregation was monitored in situ using the established Thioflavin T (ThT) fluorescence assay [82,83]. As can be seen in Figure 2a, the ThT fluorescence intensity began increasing from the very beginning of the experiment, indicating a rather short lag time of hIAPP aggregation. Nevertheless, at around 90 min of incubation, the increase in intensity becomes more rapid until a plateau-like saturation regime is reached at around 180 min. The rather large variations within and between the individual ThT fluorescence measurements (see Figure S1) can be attributed to the fact that these measurements were performed without intermediate shaking in order to mimic the conditions during incubation on the sample surfaces. Under such static conditions, the diffusion of larger aggregates and aggregate clusters through the optical path may result in strong intensity fluctuation. 

The AFM image in Figure 2b shows hIAPP aggregates obtained after 30 min incubation in bulk solution. Despite this relatively short incubation time, Figure 2b reveals the formation of amyloid fibrils several hundred nanometers in length that are clustered together. The aggregate cluster also features larger particles up to about 50 nm in height, which most likely represent amorphous aggregates. For the longer incubation time of 210 min, the AFM image in Figure 2c reveals that these amorphous aggregates grew in size, with heights reaching up to almost 200 nm. All the AFM images recorded after 210 min incubation are dominated by such large amorphous aggregates and show only few amyloid fibrils (see Figure S2), which is quite remarkable considering the high ThT intensity that is observed in Figure 2a at this incubation time. Since amorphous aggregates are generally considered ThT negative [84], this indicates that the fibrils mostly formed large clusters, which are not yet segregated but are, nevertheless, difficult to find by AFM, since it records only small snapshots of the entire substrate surface.

### 2.3. hIAPP Aggregation in Contact with Flat and Nanopatterned Surfaces

**Figure 3:**
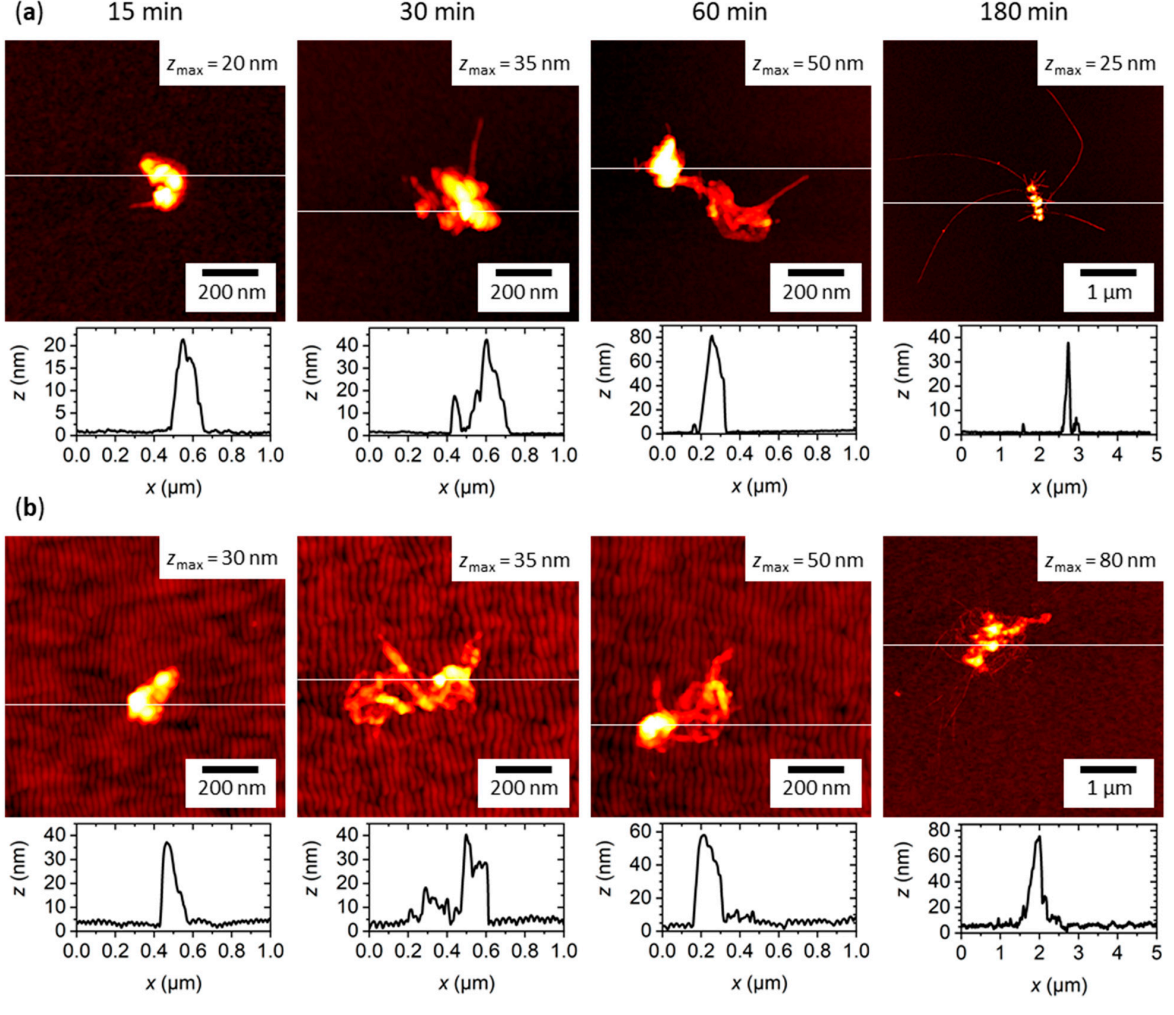
Representative AFM images of hIAPP aggregates obtained after incubation for 15 to 180 min on the different surfaces with corresponding height profiles recorded along the white lines. (**a**) Flat silicon surface with native surface oxide. (**b**) Nanopatterned silicon surface with native surface oxide. The height scales range from 0 nm to *z*_max_, with *z*_max_ given in the images. Note the different image sizes for 180 min incubation. See Figures S3–S6 for additional AFM images.

The fabricated surfaces were incubated with 2 µM hIAPP in PBS at room temperature for different times ranging from 15 to 180 min. Figure 3 shows representative AFM images of the obtained aggregates at flat (Figure 3a) and nanopatterned surfaces (Figure 3b). As can be seen, aggregates are already found on both surfaces after incubation for 15 min. It should be noted that such aggregates are observed only for incubation in hIAPP-containing PBS, not in peptide-free solution. These aggregates are rather compact and lack any discernible microstructure, suggesting that they are amorphous. With increasing incubation time, the aggregates become larger in terms of both height and lateral spread, as is particularly obvious from the height profiles displayed below the AFM images in Figure 3. Furthermore, they clearly develop a more detailed microstructure. In particular, it appears that the amorphous aggregates act as seeds for the formation of amyloid fibrils, which tend to grow outward from a central amorphous core. This is particularly obvious for the longest incubation time of 180 min, for which very long fibrils with lengths exceeding one micron are observed. 

Comparing the AFM images shown in Figure 3a,b, subtle differences in the overall shape of the hIAPP aggregates obtained at the different surfaces can be identified. While the amorphous aggregates seem rather similar in size and overall shape, the amyloid fibrils look somewhat different in appearance. In particular, at the flat surface, the amyloid fibrils are rather straight and appear to grow radially from a central amorphous core. At the nanopatterned surface, on the other hand, the fibrils are more entangled and seem to connect different amorphous aggregates. This is particularly obvious at the longest incubation time of 180 min. 

**Figure 4:**
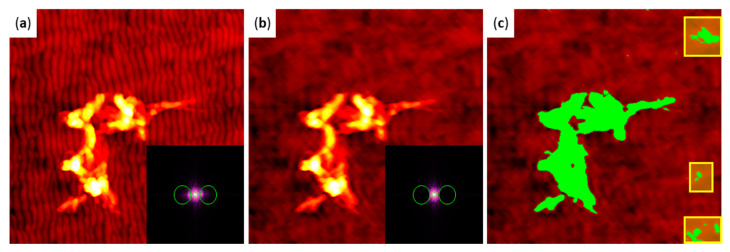
Image processing strategy employed for the quantitative analysis of aggregate morphology. (**a**) Example AFM image of hIAPP aggregates on a nanopatterned surface. The inset shows the FFT, with the green circles indicating the correlation peaks characteristic for the ripple pattern. (**b**) Filtered AFM image after subtraction of the areas in the FFT indicated by the green circles (inset). The ripple pattern in the background has mostly vanished. (**c**) Same image as in (**b**) after applying a height threshold of 8.2 nm and masking all topographic features above the threshold (green). Note that some topographic artefacts that do not represent hIAPP aggregates are masked as well, particularly at the right edge of the image (highlighted by the yellow frames). Such artefacts have been excluded from further analysis.

In order to quantify the observed differences in aggregate morphology, we measured the volumes and boundary lengths of the observed hIAPP aggregates. For flat substrate surfaces, this is straightforward and can be achieved by considering only topographic features above a certain height threshold that is adjusted to cover the topographic background of the substrate [39]. In the presence of pronounced height variations such as the ripple patterns, however, this approach may result in non-negligible artefacts because it requires a rather large threshold that may cut off some portions of the aggregates. In order to minimize the impact of such artefacts, we employed Fourier filtering [85] of the AFM images to subtract the spatial frequencies associated with the ripple pattern from the overall topography. This is demonstrated in Figure 4. A topographic AFM image of an hIAPP aggregate on a nanopatterned surface is shown in Figure 4a. The inset displays the corresponding FFT. The green circles in the FFT mark the regions that correspond to the ripple pattern and have been subtracted during filtering. The result of this subtraction is shown in Figure 4b. The ripple pattern has mostly vanished and only the long-range roughness remains, which cannot be subtracted as easily due to its random nature. To accommodate this long-range roughness, a height threshold of 8.2 nm was chosen. In Figure 4c, all topographic features above this threshold are masked. The applied mask almost entirely covers the hIAPP aggregates. However, some topographic features in the background have also been masked (highlighted in yellow in Figure 4c), as well as some smaller regions and isolated pixels in the upper half of the AFM image. These artefacts had to be excluded from further analyses by manually correlating the automatically obtained volume and boundary length values with the hIAPP aggregates visible in the AFM images.

**Figure 5:**
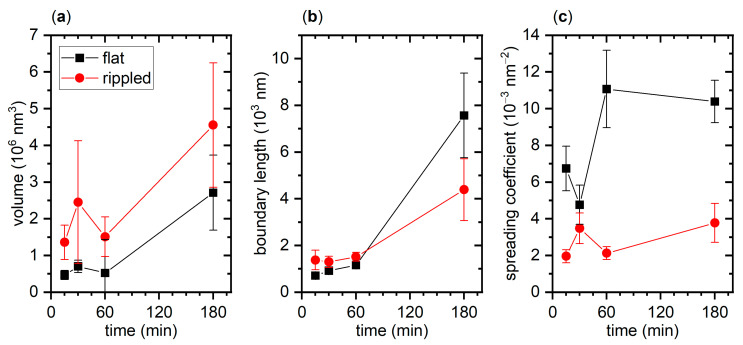
Results of the quantitative analysis of the AFM images. (**a**) Aggregate volume. (**b**) Boundary length. (**c**) Spreading coefficient. Each data point represents the average of several individual aggregates (17 ≤ *N* ≤ 47) obtained in two independent experiments. Error bars represent the standard error of the mean.

The volume and boundary length values of the individual aggregates obtained for a given surface and incubation time were averaged and are displayed in Figure 5a,b, respectively. From these data, it becomes clear that nanoscale surface topography affects both aggregate properties. In particular, Figure 5a reveals that the aggregates obtained at the nanopatterned surface have, on average, a larger volume than the ones at the flat surface do. This is observed at all incubation times, including the shortest one. Since the aggregate volume is dominated by the comparatively large amorphous aggregates, this indicates that the nanopatterned surface promotes the formation of amorphous hIAPP aggregates. Furthermore, the average volume shows a similar dependence on incubation time for both surfaces, which hints at similar kinetics of amorphous hIAPP aggregation.

A different behavior is observed for the average aggregate boundary length (see Figure 5b). Here, both surfaces behave rather similarly at short incubation times of up to one hour, which agrees well with the qualitative evaluation of the AFM images in Figure 3. For 180 min incubation, however, the average boundary length obtained for the flat surface is almost twice as long as that of the nanopatterned one. This indicates that the hIAPP aggregates that formed at the nanopatterned surface are more compact in shape, despite having a larger volume. This again agrees with the visual inspection of the AFM images in Figure 3, which revealed a stronger bundling and entanglement of the assembled fibrils. However, it should be noted that the boundary length is much more sensitive to the application of the height threshold than the aggregate volume. This is because cutting off a single long fibril from an otherwise compact and mostly amorphous aggregate by the height threshold will result in a drastically reduced boundary length but only miniscule variations in aggregate volume. Therefore, we computed the spreading coefficient defined as the ratio of boundary length and volume for each aggregate to obtain a more reliable measure that incorporates both parameters [39]. As can be seen in Figure 5c, a much larger average spreading coefficient is obtained for the flat than for the nanopatterned surface, which means that the hIAPP aggregates at the flat surface are less compact and more frayed. Furthermore, while the spreading coefficient determined for the nanopatterned surface is mostly independent of incubation time, the one for the flat surface shows a rapid increase between 30 and 60 min. This is indicative of a morphological transition and, thus, a change in the growth mode of the aggregates. We thus assume that this marks the point at which so many β sheet-rich nuclei have formed that hIAPP fibrillization becomes a dominant aggregation pathway that is at least as strong as amorphous aggregation and transforms the morphology of the aggregates from a mostly particle-like to a frayed, star-like shape. The fact that such a transition is absent or at least much weaker for the nanopatterned surface suggests that amorphous aggregation is the only dominant aggregation pathway within the full 180 min of incubation at this surface.

## 3. Discussion

A molecular model of the surface-catalyzed assembly of proteins into amyloid aggregates was recently presented by Pan et al. [86]. In this model, amyloid formation at a given surface is initiated by non-specific monomer adsorption, which results in a locally increased monomer concentration in the close vicinity of the surface compared to bulk solution. This was predicted to lead to an increased oligomerization rate at the surface and, thus, to faster aggregation. This behavior was also verified experimentally for two different proteins in contact with mica surfaces [86]. In contrast to this prediction, however, the results presented in this paper indicate that hIAPP aggregation at the silicon oxide surface is slower than in bulk solution. This is quite surprising, since it is rather well established that negatively charged interfaces promote hIAPP aggregation and fibrillization [87,88,89]. Silicon oxide has an isoelectric point below four [90] and is, thus, negatively charged at pH 7.4. However, under the physiological conditions used in our experiments, silicon oxide has a rather low surface charge density of about −0.05 C/m^2^ [63]. It was shown previously for negatively charged lipid bilayers that hIAPP aggregation proceeds more rapidly at higher charge densities [88]. We therefore assume that the electrostatic interactions between the positively charged peptide and the slightly negatively charged surface are too weak to result in a significantly increased monomer concentration and thereby an accelerated amyloid aggregation. On the contrary, it appears that the presence of the surface leads to a delay in aggregation. This is in agreement with the Monte Carlo simulations by Co and Li, which revealed that the presence of a weakly adsorbing surface may indeed slow down peptide aggregation [24]. For this type of surface, the authors also observed that increasing the surface roughness led to a further delay in aggregation. While in our experiments, this might, to some extent, be the case when considering only hIAPP fibrillization, the whole scenario appears more complex.

In particular, we observed that hIAPP aggregation at silicon oxide surfaces involves two types of aggregates, i.e., amyloid fibrils and amorphous aggregates. In particular, amorphous aggregates were already observed after incubation for only a few minutes, while fibrils appeared later and usually in association with the amorphous aggregates (see Figure 3). Such amorphous-nucleated fibril growth was previously observed for the light-chain amyloidosis-associated protein SMA in contact with negatively charged mica surfaces, but not in the presence of positively charged or hydrophobic surfaces [59]. Most notably, this type of growth occurred under conditions that resulted exclusively in amorphous aggregates in bulk solution. This is different from the present experiments with hIAPP, where similar aggregates composed of amorphous particles and amyloid fibrils were also observed in bulk (see Figure 2b), indicating that the overall pathway of aggregation is not affected by the presence of the flat silicon oxide surface. 

The role of amorphous aggregates in amyloid fibrillization is still a matter of debate [91]. In general, amorphous aggregation may compete with amyloid fibrillization or represent an intermediate step within the amyloid fibrillization pathway. The latter case is thought to involve the secondary nucleation of amyloid fibrils either inside or at the surfaces of the amorphous aggregates. Based on the AFM images of the hIAPP aggregates formed both in bulk solution and at the flat and nanopatterned silicon oxide surfaces, secondary nucleation of fibrils by amorphous aggregates seems to be the dominant mechanism of hIAPP fibrillization in our experiments (see Figure 2 and Figure 3). Thus, it appears reasonable that the amorphous aggregates that formed in contact with the nanopatterned surface exhibit a different structure that lowers their propensity for nucleating amyloid formation. In the case of surface-mediated nucleation, this would imply a different surface structure of the amorphous aggregates with fewer nucleation sites. In the case of internal nucleation, changes in the density of and the monomer mobility inside the amorphous phase may result in reduced amyloid aggregation [91].

While our observations are qualitatively in line with those of Shezad et al. [23], there are some distinct differences. For instance, the authors investigated Aβ(1–42) aggregation at random rough PS surfaces (*S*_q_ between 0.26 and 1.81 nm) at a monomer concentration below the critical concentration for fibril formation in bulk solution. Using AFM, they observed a variety of oligomeric, fibrillar, and amorphous aggregates developing at the flat surface within 6 h of incubation. At the rough surface, however, they found only oligomers and amorphous aggregates, with no fibrils at all. Based on single-molecule trajectories measured by total internal reflection fluorescence (TIRF) microscopy, they attributed the observed inhibition of peptide fibrillization to the reduced lateral diffusion of adsorbed peptide monomers at the rough surfaces. In our experiments, we did not observe complete inhibition of fibrillization but rather a retardation at the expense of more pronounced amorphous aggregation. This is most likely because the monomer concentration was above the threshold for bulk fibrillization; as such, aggregation may also involve peptide monomers and oligomers from bulk solution with both amorphous aggregates and amyloid fibrils growing away from the surface into solution.

**Figure 6:**
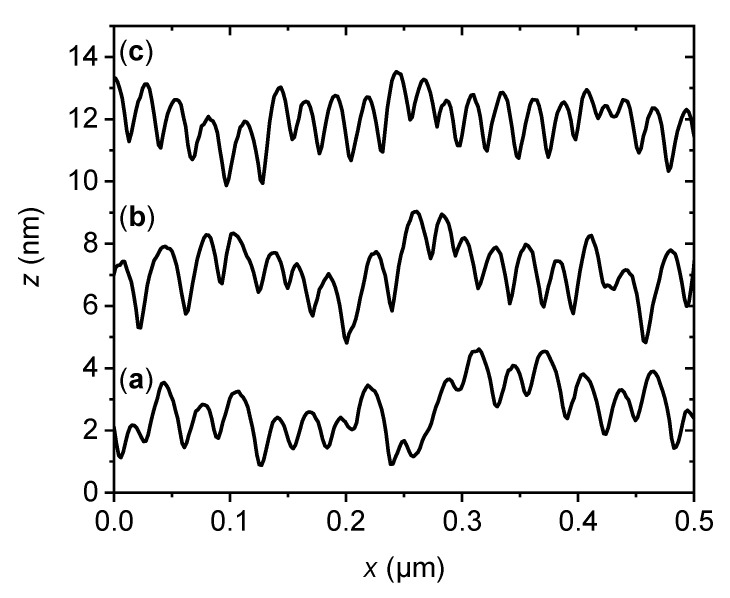
Representative height profiles taken across the ripple patterns on a nanopatterned silicon oxide surface (**a**) before and (**b**,**c**) after incubation with hIAPP for (**b**) 60 and (**c**) 180 min. The profiles are shifted vertically for clarity.

Furthermore, even though the nanopatterned silicon oxide surfaces employed in our current experiments had similar *S*_q_ values as the random rough PS surfaces used by Shezad et al., i.e., about 1.2 nm, the morphologies differed quite drastically. In particular, the nanorippled surfaces exhibit a pronounced anisotropy (see FFT in Figure 1b). One would thus imagine that the diffusion of adsorbed peptides also becomes anisotropic with a longer average diffusion length along the ripples, resulting in preferential fibril growth in this direction. However, we did not see any indication that the assembled fibrils aligned along the ripples. There are a few possible explanations that might account for this lack of fibril alignment. First, one could imagine that adsorption of peptide monomers may, over time, smooth out the ripple pattern, as previously observed for serum albumin adsorption at nanorippled titanium oxide surfaces [15]. However, as can be seen in Figure 6, the topographic height modulation of the nanorippled silicon oxide surface was barely altered during prolonged incubation with hIAPP-containing PBS solution. We can, thus, rule out this possibility. Second, the isotropic long-range roughness overlaying the ripple pattern may also restrict surface diffusion in the direction parallel to the ripples. Even though this long-range roughness acts on much larger length scales of hundreds of nanometers [92,93], Shezad et al. have shown that Aβ(1–42) monomers adsorbed at a flat PS surface diffuse over distances of a few microns within seconds. However, the ripple pattern still represents a very dominant topographic feature in the sub-100-nanometer range (see Figure 6) and should, thus, present an additional barrier to peptide diffusion in this direction. We would therefore expect to observe at least some degree of alignment of the peptide aggregates. Since this is not the case, we consider the lack of any fibril alignment as one more indication that fibril growth involves mostly non-adsorbed peptide monomers and oligomers from bulk solution.

## 4. Materials and Methods

### 4.1. Substrate Preparation

The epi-ready, p-doped Si(100) wafers employed as substrates were purchased from Siegert Wafer (Aachen, Germany). The wafers were cut into 1 × 1 cm^2^ pieces and cleaned for 15 min at 75 °C in fresh RCA1 solution (1:1:5 NH_4_OH:H_2_O_2_:H_2_O). Ion beam nanopatterning was performed as previously described [15] using a Kaufman-type KDC 40 ion source (Kaufman & Robinson Inc., Fort Collins, CO, USA) mounted in a vacuum chamber with a base pressure of about 1 × 10^−7^ mbar. Irradiation was performed with 500 eV Ar^+^ at an incident angle of 67° with respect to the surface normal. The applied flux and fluence were 2 × 10^14^ cm^−2^s^−1^ and 1 × 10^17^ cm^−2^, respectively. Immediately before incubation with hIAPP, all substrates were treated with oxygen plasma (diener Zepto, Diener electronic, Ebhausen, Germany) for 30 s to achieve a reproducible highly oxidized surface chemical state.

### 4.2. Peptide Preparation 

The hIAPP stocks were prepared as previously described [39]. To this end, 1 mg of hIAPP (BACHEM AG, Bubendorf, Switzerland) was dissolved in 512 µL 1,1,1,3,3,3-hexafluoro-2-propanol (HFIP, Thermo Fisher, Kandel, Germany) and stored for 1 h at room temperature with occasional vortexing. After centrifugation at 15,000 rpm in a microcentrifuge (VWR International, Darmstadt, Germany), the upper ~80% of the solution was divided into 30-microliter aliquots. HFIP was evaporated overnight in a fume hood to obtain the dried peptide films. After HFIP evaporation, the dried aliquots were stored at −21 °C until further use. Since the current experiments required comparatively small amounts of hIAPP, the dry peptide films were redissolved in 75 µL HFIP each, vortexed for 30 s, centrifuged for 15 s using an Eppendorf MiniSpin centrifuge (Eppendorf, Hamburg, Germany), and divided into 10-microliter aliquots. These 10-microliter aliquots were dried again as described above and stored at −21 °C. Immediately before each experiment, a dried hIAPP aliquot was slowly brought to room temperature and subsequently dissolved in 10 µL dimethyl sulfoxide (DMSO, Sigma-Aldrich, Steinheim, Germany), vortexed for 30 s, centrifuged for 15 s, and allowed to reach equilibrium for 10 min.

### 4.3. hIAPP Aggregation in Bulk Solution

To investigate hIAPP aggregation in bulk solution by ThT fluorimetry, a fresh stock solution of ThT (Sigma-Aldrich, Steinheim, Germany) in PBS (Sigma-Aldrich, Steinheim, Germany), containing 138 mM sodium chloride, 2.7 mM potassium chloride, 10 mM sodium phosphate, and 2.7 mM potassium phosphate at pH 7.4, was prepared before each measurement. To this end, 2.8–8 mg of ThT was solved in 2 mL HPLC-grade water (VWR International, Fontenay-sous-Bois, France) and ultrasonicated for 15 min. The solution was then filtered using a syringe filter with 0.2 µm PTFE membrane (VWR International, Darmstadt, Germany) and diluted in HPLC-grade water at a ratio of 1:10. The concentration of ThT was calculated from its absorbance at 412 nm as measured with an Implen 330 UV/VIS nanophotometer (Implen GmbH, München, Germany), using an extinction coefficient of 36,000 M^−1^ cm^−1^. This solution was mixed with PBS to obtain a ThT concentration of 20 µM. Then, 5 µL of DMSO-hIAPP solution was added to 495 µL of PBS-ThT solution, resulting in a final hIAPP concentration of 2 µM. The solution was vortexed for 15 s at 2800 rpm and then added to a quartz cuvette (Hellma GmbH & Co. KG, Müllheim, Germany). The measurements were performed at room temperature using a JASCO fluorospectrometer FP-8200 (JASCO Deutschland GmbH, Pfungstadt, Germany) and an excitation wavelength of 440 nm. The ThT fluorescence emission intensity at 485 nm was recorded in 60-second intervals over a time course of 5 h. The experiment was repeated twice, and the curve shown in Figure 2a represents the average of the three resulting curves (see Figure S1).

To investigate hIAPP aggregation in bulk solution by AFM, 4 µL of DMSO-hIAPP solution was added to 396 µL of PBS and subsequently vortexed for 15 s at 2800 rpm. Then, 400 µL of 2 µM hIAPP solution was incubated in a microcentrifuge tube at room temperature. For each incubation time of 30 and 210 min, two 50-microliter samples were removed from the tube and deposited on two freshly cleaved mica substrates (Ted Pella, Inc., Redding, CA, USA). Mica has an atomically flat surface with a large negative surface charge density [94] and is, thus, ideally suited for immobilizing the positively charged hIAPP aggregates formed in solution [39,50,55,60]. After incubation for an additional 15 min, each mica surface was washed with about 4 mL of HPLC-grade water and dried in a stream of ultra-pure air.

### 4.4. hIAPP Aggregation in Contact with Flat and Nanopatterned Surfaces

To investigate hIAPP aggregation in contact with the flat and nanopatterned surfaces, 4 µL of the DMSO-hIAPP solution was added to 396 µL PBS to yield a final peptide concentration of 2 µM and subsequently vortexed for 15 s at 2800 rpm. Then, 100 µL of this hIAPP solution was incubated on the flat and nanopatterned surfaces (1 × 1 cm^2^) at room temperature in a humidity cell with a water reservoir to avoid evaporation of the peptide sample. After incubation for different times ranging from 15 to 180 min, samples were removed from the cell, gently washed with HPLC-grade water, and dried in a stream of ultra-pure air. For the nanopatterned surfaces, washing was performed at an azimuthal angle of about 45° with respect to the direction of the ripple patterns.

### 4.5. AFM Imaging and Image Analysis

The substrate surfaces were imaged in intermittent contact mode in air using a JPK Nanowizard III AFM (JPK Instruments, Berlin, Germany) and MikroMasch HQ:NSC18/Al BS cantilevers (75 kHz, 2.8 N/m, NanoAndMore, Wetzlar, Germany). All AFM images were analyzed using Gwyddion open source software version 2.56 [95]. Fourier filtering of the flattened images of the nanopatterned substrates was performed using the 2D FFT Filtering tool. Aggregate volumes and boundary lengths were determined by applying a height threshold to the flattened and—for the nanopatterned substrates—filtered images using the Mark by Threshold tool. The value of the threshold was adjusted individually to exclude as much of the substrate surface as possible without compromising the measurements of the aggregate morphology. The zero-basis volumes and projected boundary lengths of all the masked areas were then extracted using the Grain Distributions tool. Since these extracted values occasionally still included substrate artefacts (see Figure 4), only those that could unambiguously be attributed to peptide aggregates were selected for further analysis.

## 5. Conclusions

In summary, we have studied the aggregation of hIAPP in contact with flat and nanorippled silicon oxide surfaces. We found that hIAPP aggregation under the employed conditions proceeds via the initial formation of amorphous aggregates, which then nucleate the growth of amyloid fibrils. The presence of a flat silicon oxide surface slowed down hIAPP aggregation but did not alter the pathway of aggregation. In contact with the anisotropic nanorippled surface, however, we observed a further retardation of hIAPP fibrillization at the expense of more pronounced amorphous aggregation. Our results suggest that under the employed conditions, hIAPP aggregation at the silicon oxide surface proceeds mostly via addition of monomers and oligomers from bulk solution. The presence of the nanopatterns most likely leads to an alteration of the internal or surface structure of the amorphous aggregates, which results in a lower propensity for nucleating amyloid fibrillization. Our results thus demonstrate that nanoscale surface topography may affect peptide and protein aggregation pathways in complex and intricate ways beyond simple retardation or promotion.

## Data Availability

The data presented in this study are available on request from the corresponding author.

## Supplementary Materials

The following are available online at https://www.mdpi.com/article/10.3390/ijms22105142/s1, Figure S1: ThT fluorescence measurements of hIAPP aggregation in bulk solution; Figure S2: AFM images of hIAPP aggregates obtained after 30 min and 60 min incubation in bulk solution without ThT; Figure S3: AFM images of hIAPP aggregates obtained after 15 min incubation in contact with the flat and nanorippled surfaces; Figure S4: AFM images of hIAPP aggregates obtained after 30 min incubation in contact with the flat and nanorippled surfaces; Figure S5: AFM images of hIAPP aggregates obtained after 60 min incubation in contact with the flat and nanorippled surfaces. Figure S6: AFM images of hIAPP aggregates obtained after 180 min incubation in contact the flat and nanorippled surfaces.
