# Supplementary material for: Nanoscale Surface Topography Modulates hIAPP Aggregation Pathways at Solid–Liquid Interfaces
Source: Int J Mol Sci. 2021 May 13;22(10):5142. doi: 10.3390/ijms22105142 (PMC8152259; doi:10.3390/ijms22105142)
Supplement: Supplementary file 1 [file ijms-22-05142-s001.zip › ijms-1211696-supplementary.pdf]

# Supplementary Materials

# Nanoscale Surface Topography Modulates hIAPP Aggregation Pathways at Solid–Liquid Interfaces

*Marcel Hanke, Yu Yang, Yuxin Ji, Guido Grundmeier and Adrian Keller**

Paderborn University, Technical and Macromolecular Chemistry, Warburger Str. 100, 33098 Paderborn, Germany.

* Correspondence: adrian.keller@uni-paderborn.de, Tel.: +49-5251-60-5722

**ThT fluorescence analysis of hIAPP aggregation bulk solution**

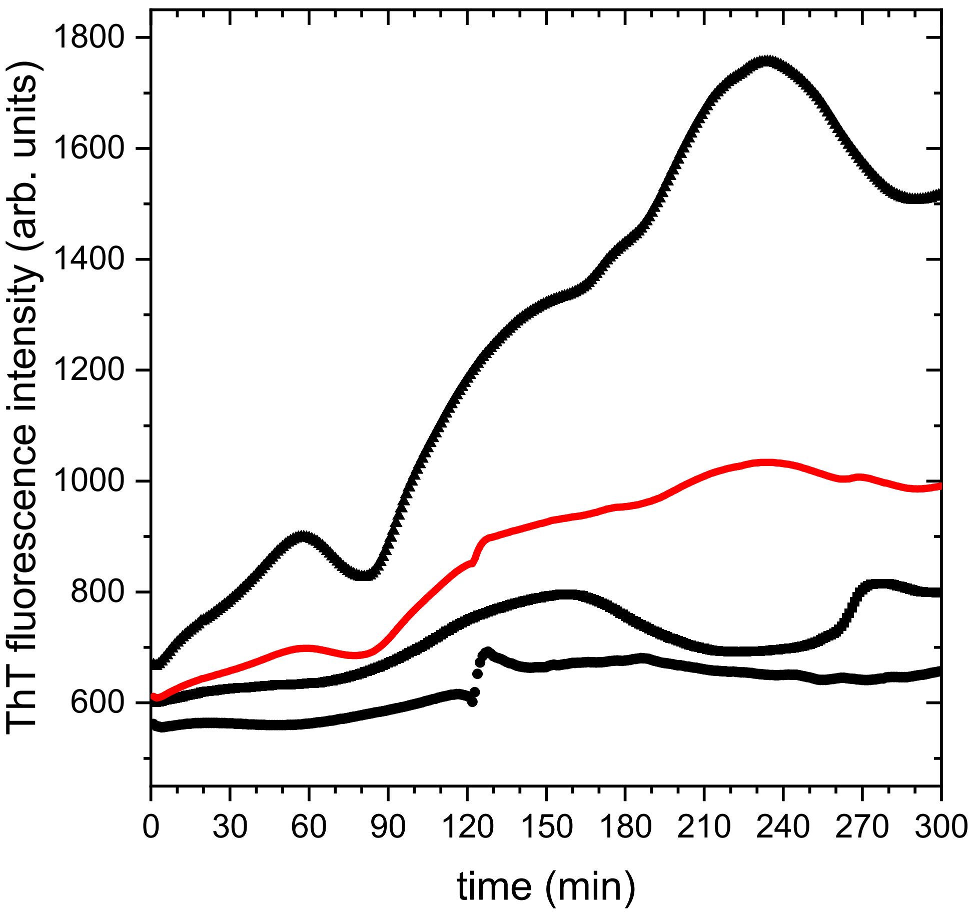


***Figure S1:*** *ThT fluorescence measurements of hIAPP aggregation in bulk solution. The black data represent three independent measurements while the red curve is their average. Even though the overall fluorescence intensity varies a lot between individual measurements, all curves show an increase in intensity between 60 and 90 min, followed with a saturation regime that is entered between 130 and 160 min.*

## Additional AFM images of hIAPP aggregates formed in bulk solution

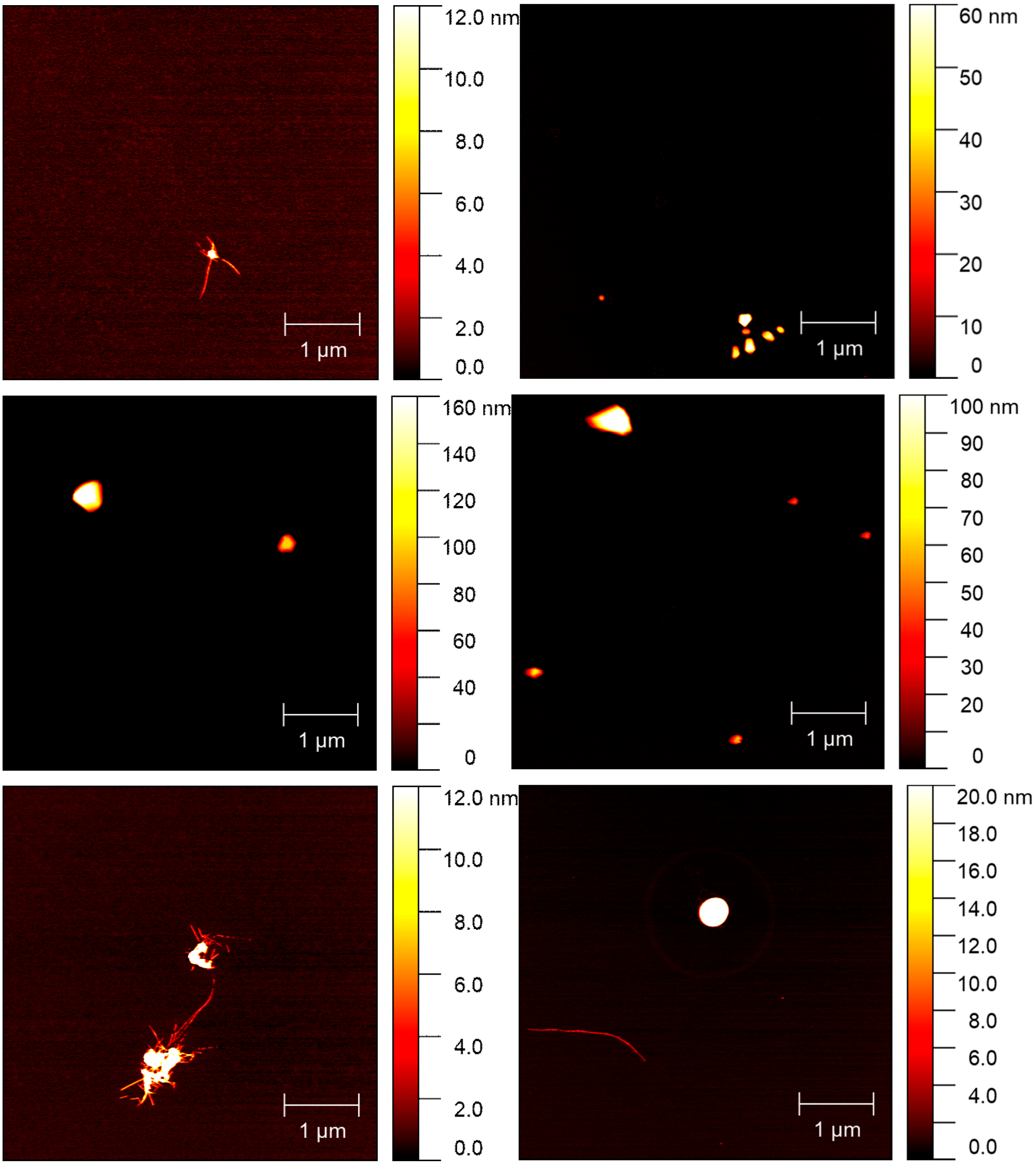


***Figure S2:*** *AFM images of hIAPP aggregates obtained after 30 min (left) and 210 min (right) incubation in bulk solution without ThT.*

**Additional AFM images of hIAPP aggregates formed at the flat and nanorippled surfaces**

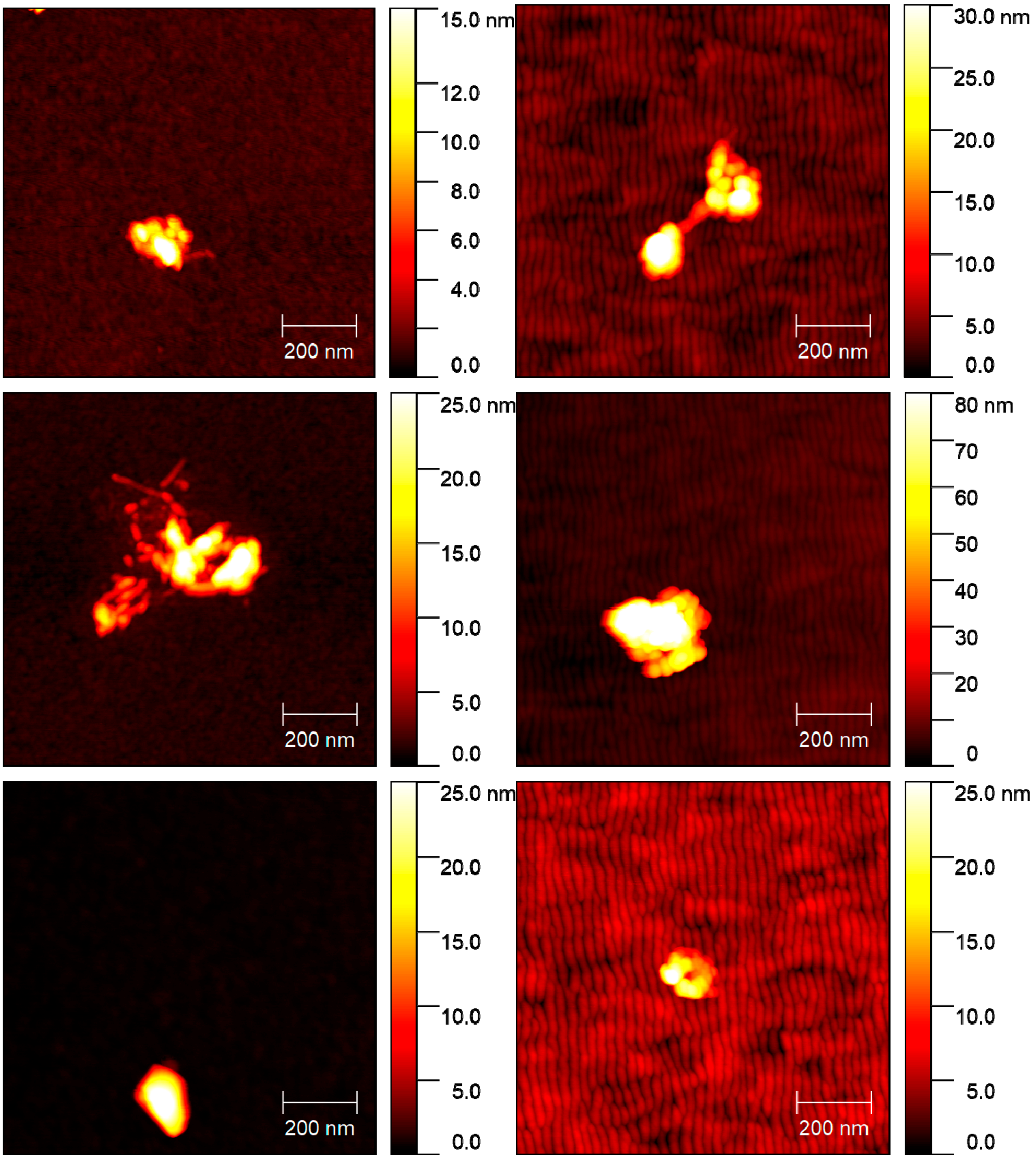


***Figure S3:*** *AFM images of hIAPP aggregates obtained after 15 min incubation in contact with the flat (left) and nanorippled (right) surfaces.*

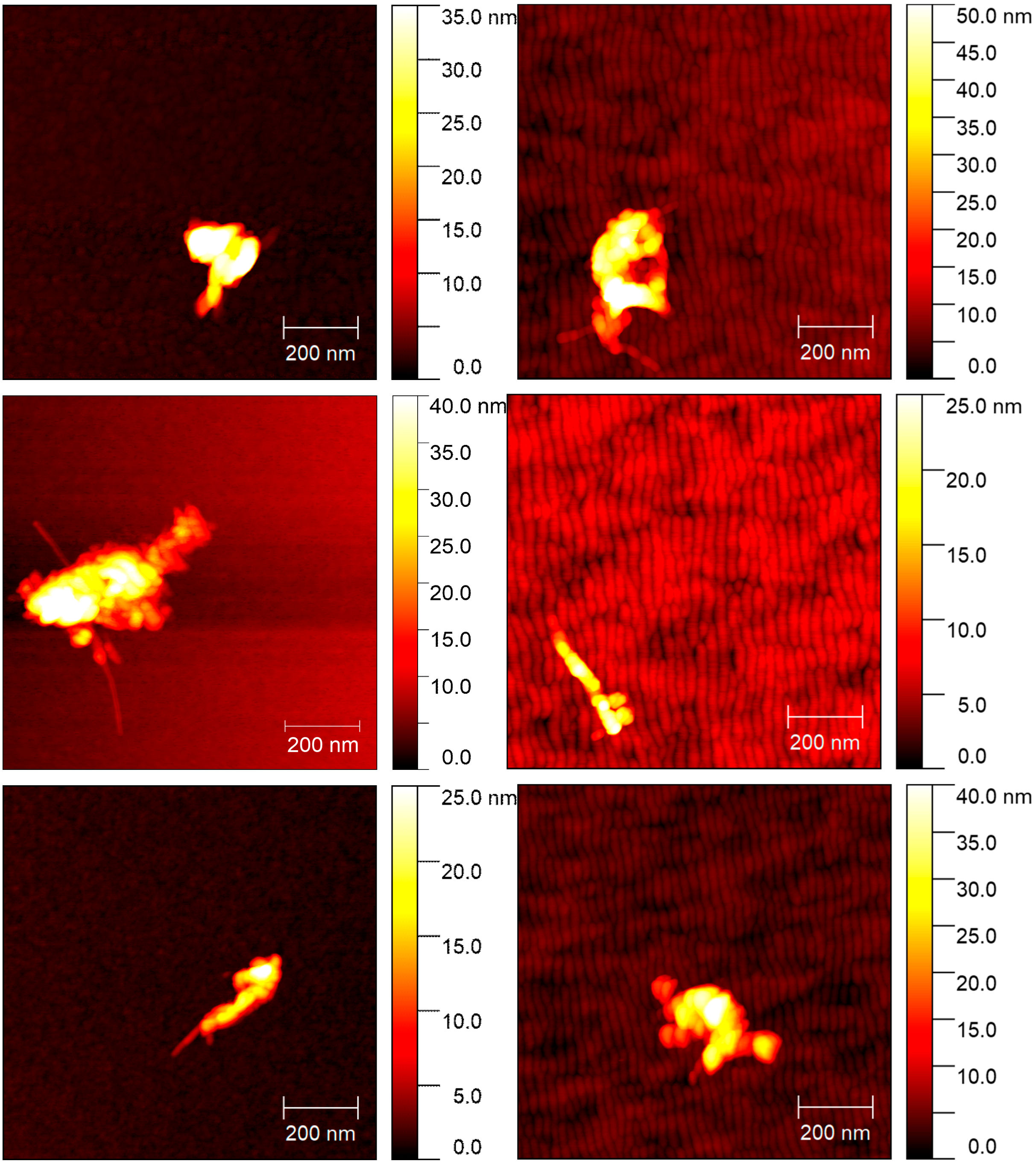


***Figure S4:*** *AFM images of hIAPP aggregates obtained after 30 min incubation in contact with the flat (left) and nanorippled (right) surfaces.*

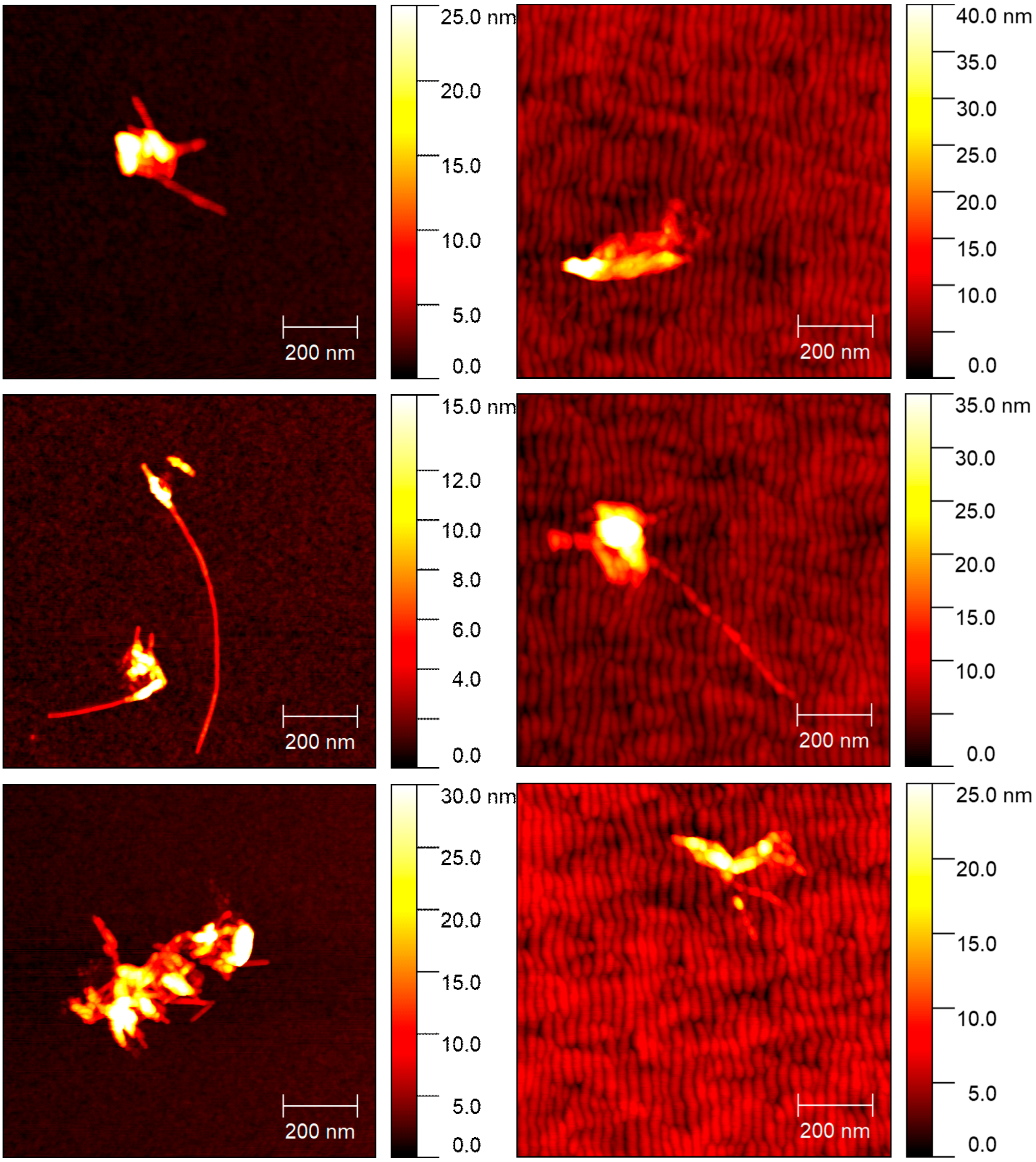


***Figure S5:*** *AFM images of hIAPP aggregates obtained after 60 min incubation in contact with the flat (left) and nanorippled (right) surfaces.*

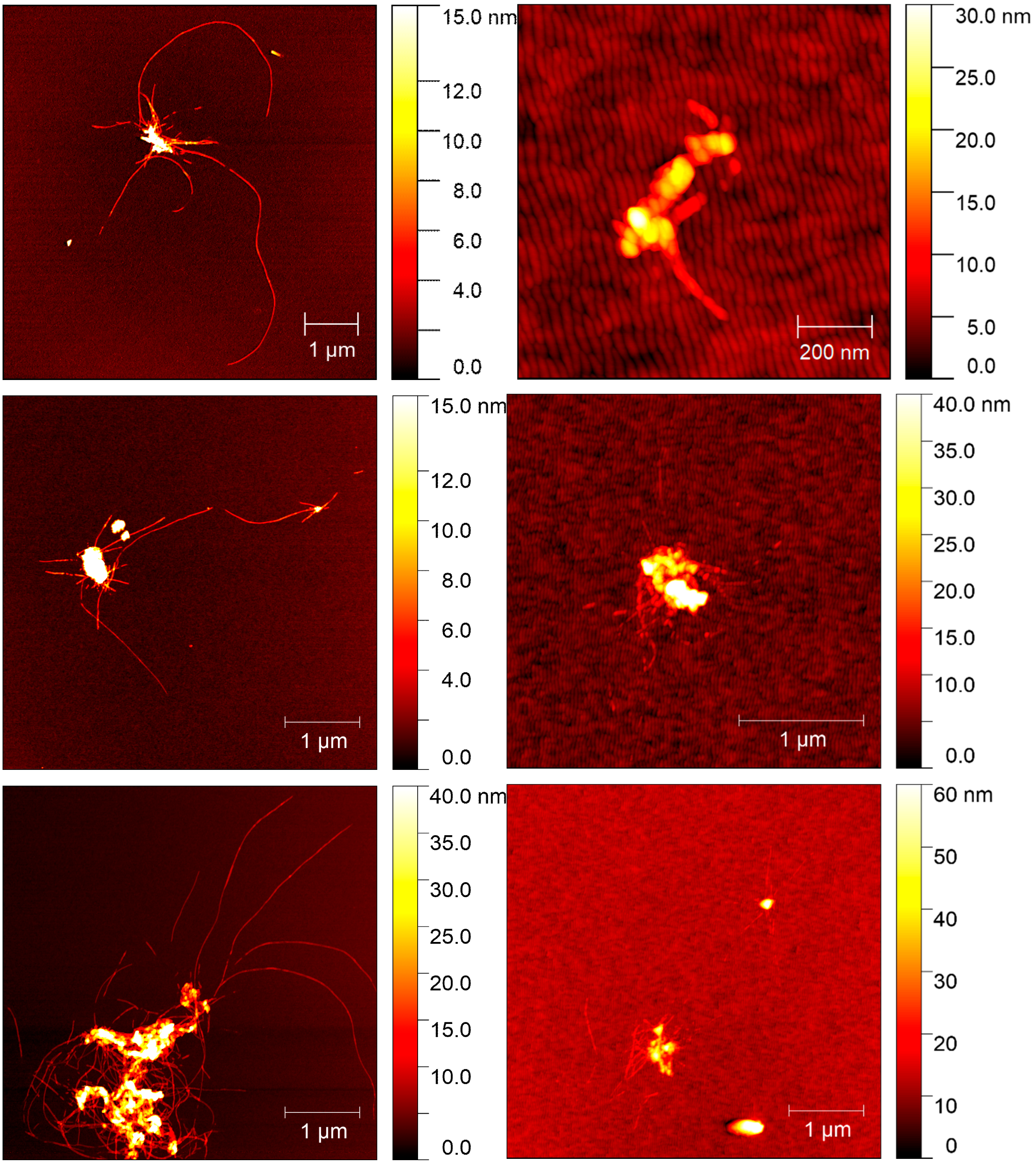


***Figure S6:*** *AFM images of hIAPP aggregates obtained after 180 min incubation in contact the flat (left) and nanorippled (right) surfaces.*
